# Celiac Disease After Administration of Immune Checkpoint Inhibitors: A Case Report

**DOI:** 10.3389/fimmu.2021.799666

**Published:** 2021-12-17

**Authors:** Julie Leblanc, Solene Hoibian, Agathe Boucraut, Jean-Philippe Ratone, Louis Stoffaes, Domitille Dano, Delphine Louvel-Perrot, Brice Chanez, Anne-Sophie Chretien, Anne Madroszyk, Philippe Rochigneux

**Affiliations:** ^1^ Medical Oncology Department, Paoli-Calmettes Institute, Aix-Marseille University, Marseille, France; ^2^ Gastroenterology Department, Paoli-Calmettes Institute, Aix-Marseille University, Marseille, France; ^3^ Pathology Department, Paoli-Calmettes Institute, Aix-Marseille University, Marseille, France; ^4^ Team Immunity and Cancer, Centre de Recherche en Cancérologie de Marseille (CRCM), Inserm, U1068, CNRS, UMR7258, Paoli-Calmettes Institute, Aix-Marseille University, Marseille, France

**Keywords:** celiac disease, immune checkpoint inhibitors, immune toxicity, digestive toxicity, nivolumab, case report

## Abstract

Immune checkpoint inhibitors (ICI) reinvigorate the immune system to recognize and destroy tumor cells. Because of this biological mechanism, patients might develop autoimmune toxicities, notably in the digestive tract (most frequently, hepatitis or colitis). A 70-year-old man with relapsed mesothelioma was treated with nivolumab in 3rd line. He was hospitalized for watery and foul-smelling diarrhea. He underwent gastrointestinal endoscopy, showing duodenitis and villous atrophy and measurement of serum IgA antibodies to tissue transglutaminase (tTG-IgA+), leading to the diagnosis of ICI-induced celiac disease. He was treated with steroids, proton pump inhibitors, and a gluten-free diet. If ICI-induced celiac disease is rare in the literature, increasing reports suggest that celiac disease might represent an underestimated ICI toxicity. This case highlights the necessity of complementary investigation (including tTG-IgA and endoscopic biopsies) in patients with atypical digestive symptoms during immunotherapy.

## Introduction

Immune checkpoint inhibitors (ICIs) enhance the ability of the patient’s own immune system to recognize and destroy tumor cells. Nivolumab is a fully human monoclonal antibody that binds PD-1 on activated immune cells and disrupts binding of PD-1 to its ligand PD-L1. This process prevents downregulation of cytotoxic T cells and increases the host antitumor response (1). However, by increasing the activity of the immune system and disinhibiting T-cell function, PD-1 inhibitors may induce inflammatory side effects, termed immune-related adverse events (irAEs). Most commonly involved systems are the gastrointestinal tract, endocrine glands, skin, and liver (2).

Among digestive toxicity, colitis and hepatitis are often described (3). Colitis presentation is mostly diarrhea, and other symptoms include abdominal pain, hematochezia, weight loss, fevers, nausea, and vomiting (4, 5). Actually, celiac disease (CeD) induced by immune checkpoint inhibitor (ICI-CeD) has the same clinical presentation, but is much less frequent, resulting in diagnosis challenges.

CeD is an autoimmune disorder characterized by a chronic small intestinal enteropathy precipitated by exposure to dietary gluten in genetically predisposed individuals (6). Ingestion of gluten leads to an overreaction of the immune system, causing inflammation and destruction of the villi of the intestinal mucosa. The classic presentation is diarrhea, weight loss, malabsorption syndrome, and abdominal pain (7). Diagnosis is based on measurement of serum IgA antibodies to tissue transglutaminase (tTG-IgA), with histopathological confirmation when available. Currently, the only treatment for CeD is a lifelong, strict gluten-free diet (8).

Here, we report a case of patient with pleural mesothelioma experiencing a CeD after 2 infusions of nivolumab.

## Case Presentation

A 70-year-old man presented shortness of breath in July 2017. He did not report any oncological or autoimmune familial medical history, but had a personal history of type 1 diabetes, dyslipidemia, and arterial hypertension. A thoracoscopy allowed pleural fluid evacuation and the diagnosis of epithelioid malignant pleural mesothelioma. Frontline chemotherapy by cisplatin-pemetrexed was started and was switched to carboplatin-pemetrexed due to deterioration of renal function (6 cycles). In November 2017, he started vinorelbine due to pleural effusion relapse.

In March 2021, as he presented an increase of dyspnea and needed several thoracentesis, CT scan showed a nodular thickening of pleura. The tumor board decided to treat him with nivolumab in 3rd line (240 mg every 2 weeks). After the 1st infusion (March 18, 2021), he presented with grade 2 asthenia, grade 1 vomiting, and gastroesophageal reflux disease (GERD) with a 3-kg weight loss. Two days after the second infusion (March 31, 2021), the patient contacted us for asthenia, vomiting, and grade 3 diarrhea, limiting his quality of life (treated at home by diosmectite, loperamide, and racecadotril). The 3rd infusion was reported by 2 weeks. He was hospitalized just before the 3rd infusion because of watery and foul-smelling diarrhea, without blood, GERD, fluctuating nausea, and vomiting, complicated by dehydration and hypotension. Physical examination revealed a grade 1 sinus tachycardia, a known pleural effusion, and a normal abdomen. Biologically, he had normal plasmatic values of ionogram, TSH, ACTH, and cortisol. The dosage of total immunoglobulins was normal, and the serum protein electrophoresis only showed an inflammatory profile. Stool culture, *Clostridium difficile* research, and parasitological examination of the stool were negative.

**Figure 1:**
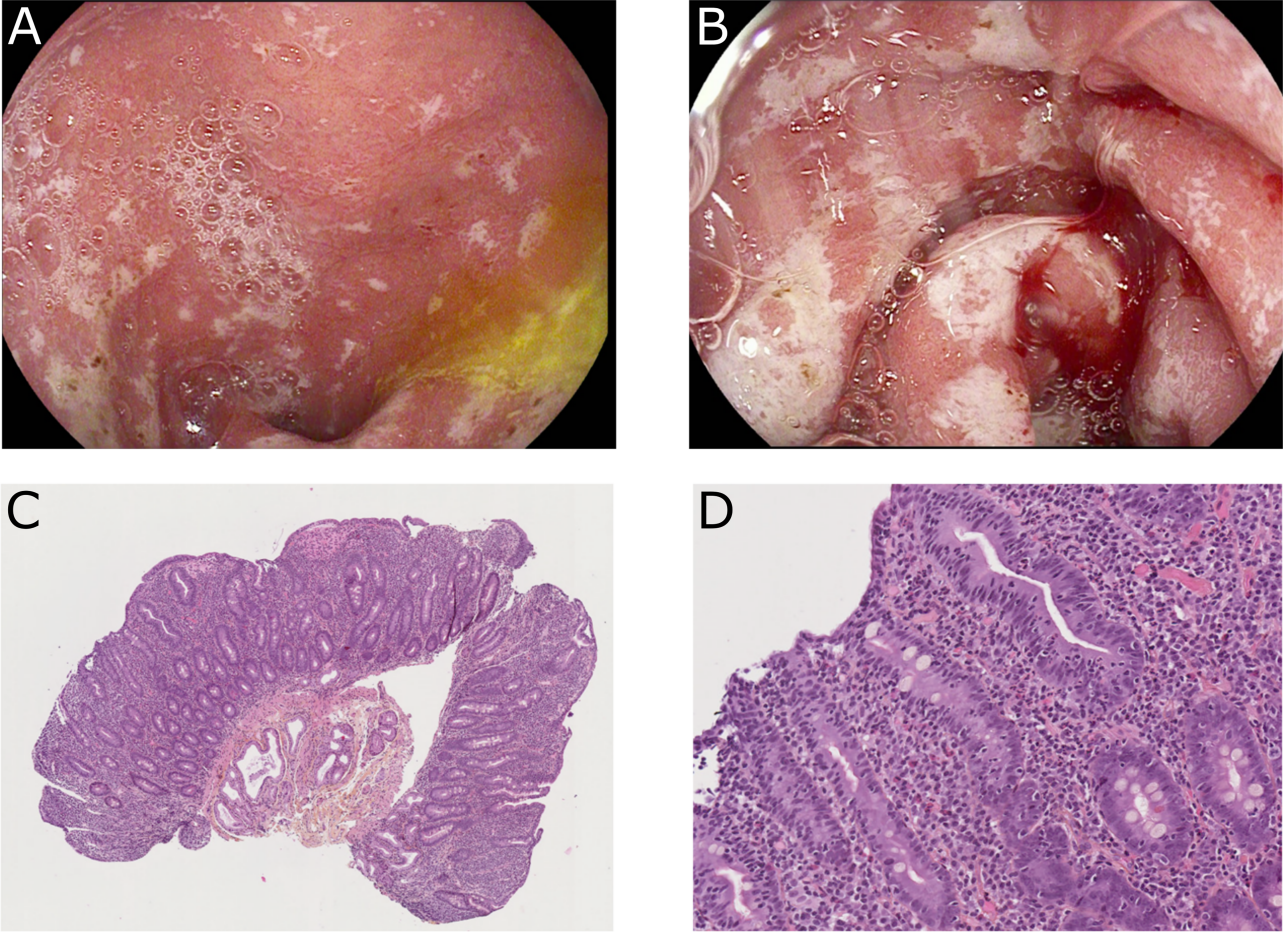
Endoscopic findings **(A, B)** and duodenal biopsies **(C, D)** after 2 cycles of nivolumab. **(A, B)** Major duodenitis associated with diffuse superficial ulcerations. **(C, D)** Histological examination revealed elementary lesions: increased intraepithelial T lymphocytes (IEL) (>30 IEL/100 enterocytes), crypt hyperplasia, and villous atrophy with alteration of normal crypt/villous ratio (3:1).

To progress toward a diagnosis, we performed endoscopic evaluation. Ileocolonoscopy with ileal and colic biopsies were normal, eliminating Crohn’s disease, ulcerative colitis, or ICI-induced colitis. Esophago-gastroduodenoscopy (EGD) showed a major duodenitis with erythematous aspect and diffuse superficial ulcerations (**Figures 1A, B**). To eliminate infectious enteropathy, we performed intestinal biopsies with normal bacteriological and virological examination. Surprisingly, histological examination revealed elementary lesions of CeD: increased intraepithelial T lymphocytes (IEL) (>30 IEL/100 enterocytes), crypt hyperplasia, marked villous atrophy, and alteration of normal crypt/villous ratio (3:1) (**Figures 1C, D**). The CeD was graded type 3b according to the modified Marsh classification of histologic findings in CeD (Oberhuber). Serum IgA antibodies to tissue transglutaminase were positive (128 U/ml, with a lab norm of 10 U/ml) and anti-endomysial IgA were positive (80 U/ml, with a lab norm of 10 U/ml). Altogether, the endoscopic, histological, and biological results led us to the diagnosis of CeD induced by immune checkpoint inhibitors (anti PD-1 nivolumab).

He was initially treated with proton pump inhibitors (PPI) intravenously 200 mg/day for 48 h and then 120 mg/day (40 mg 3 times a day). He received PPI at 80 mg for 1 month. In the face of persistent diarrhea despite anti-diarrheal medication, methylprednisolone 1 mg/kg/day was introduced initially intravenously for 3 days, then prednisolone per os at a dose of 1 mg/kg/day for 2 months, followed by a progressive decrease until 10 mg/day. A test to eliminate adrenal insufficiency was performed, before stopping corticosteroid. A gluten-free diet was also introduced in hospitalization for a long-term perspective. The patient remained in the hospital for 2 weeks and nivolumab was stopped. Ten days after the end of hospitalization, he only had three diarrheas per day.

**Figure 2:**
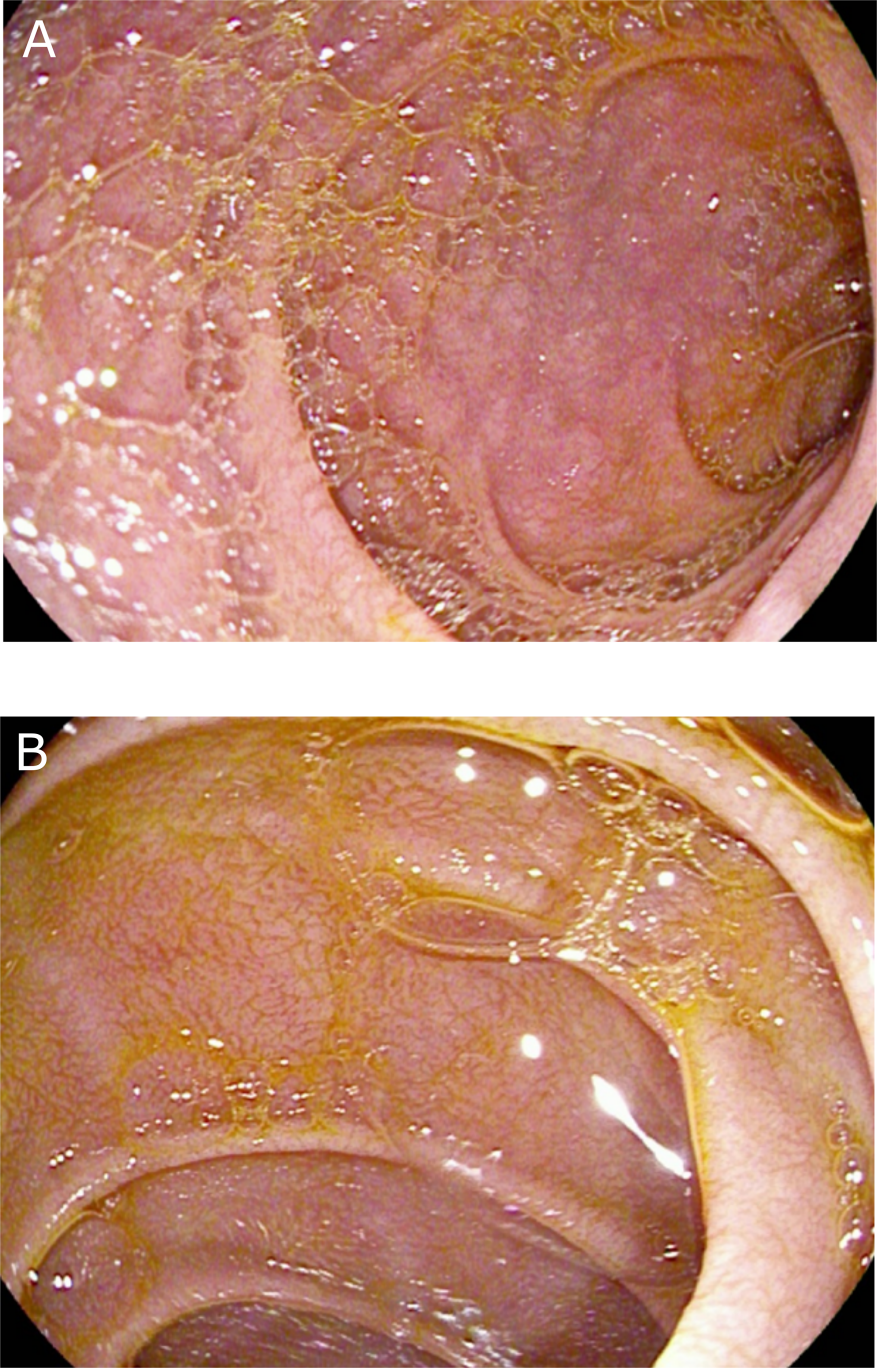
Endoscopic findings **(A, B)** after 2 months of gluten-free disease. Complete healing of the inflammatory aspect and of the ulcerations of the duodenum.

Two months later in the consultation, he mentioned a good clinical improvement and a weight gain with a good adherence of gluten-free diet. A control EGD was realized, finding a complete healing of the inflammatory aspect and of the ulcerations of the duodenum (**Figure 2**). Duodenal biopsies showed grade I villus atrophy and mild chronic aspecific gastritis (without atrophy, metaplasia, dysplasia, or *Helicobacter pylori*). A control EGD is planned 6 months later and a lifelong gluten-free diet is recommended.

**Figure 3:**
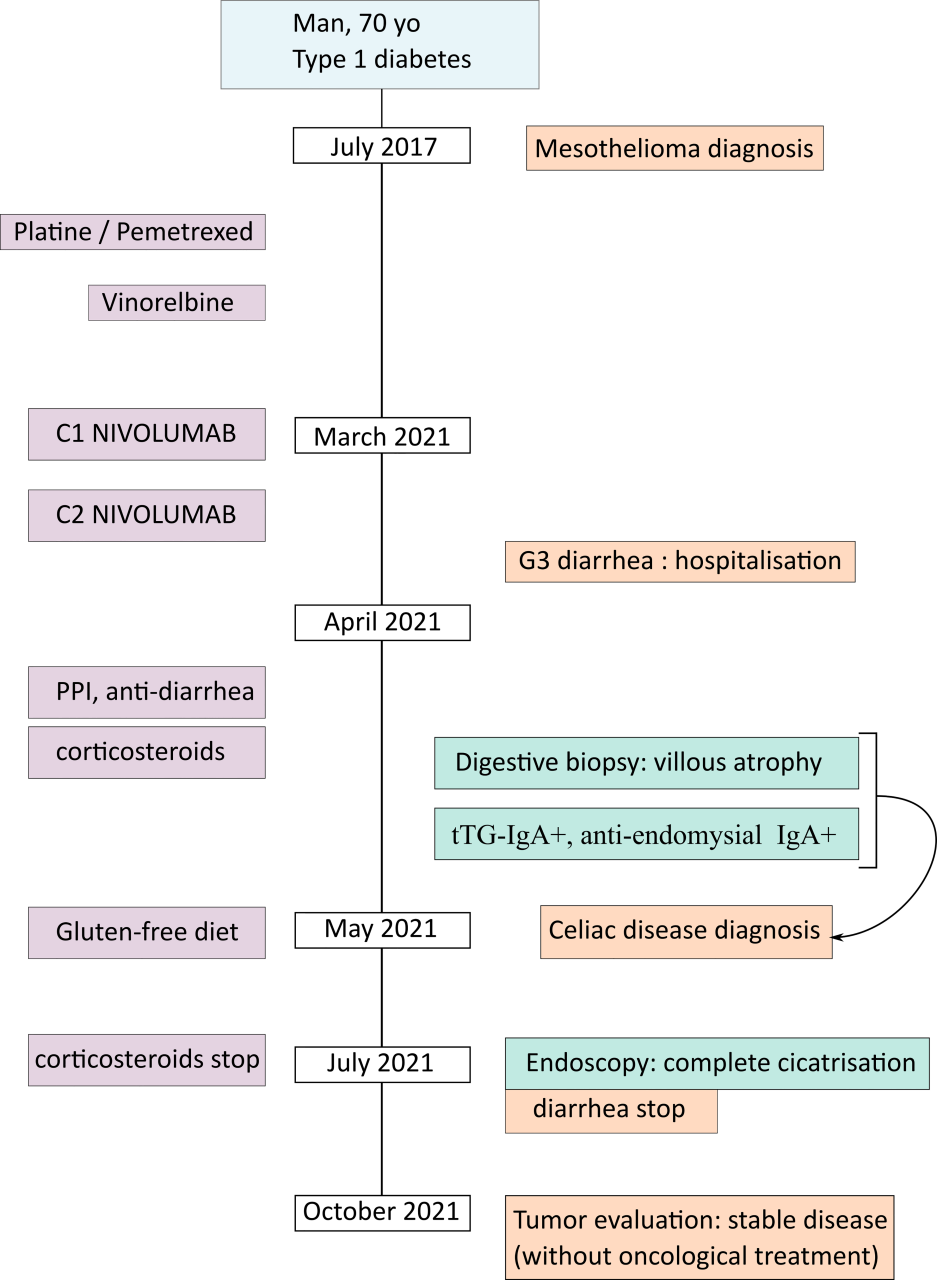
Timeline of the case report.

In an oncological perspective, 7 months after the last nivolumab infusion, the patient still had a stable disease, without requiring any additional line of systemic treatment (**Figure 3**).

## Discussion

We report here the case of a 70-year-old patient presenting with CeD induced by immune checkpoint inhibitors (anti PD-1 nivolumab). As a single-patient case report, our study has evident limitations, such as the impossibility to generalize our findings. Moreover, some specialized data, such as genetic markers for CeD (HLA subtypes) or detailed immune infiltration, are lacking. However, our aim is to alert physicians about CeD induced by ICI, a probably underestimated toxicity. Indeed, the literature only mentions 4 case reports and a case series of 8 patients receiving either anti-PD-1 or anti-CTLA-4, or both (**Table 1**). We will briefly describe these cases before discussing associated factors with ICI-CeD, physiopathology, and treatment.

**Table 1:** Published cases of patients with immune checkpoint inhibitor-induced celiac disease (ICI-CeD).

Sex, Age	ICI Name	Antibody	Endoscopic findings	Treatment	Ref.
M, 62 years old	Ipi	tTG-IgA +	Crypt apoptosis	Corticosteroid (prednisone)	Gentile et al.
		Anti-gliadin +	Crypt distortion	Infliximab	
		Anti-endomysial –	Intraepithelial lymphocytosis	GFD	
W, 63 years old	Pembro	tTG-IgA –	Villous blunting	GFD only	Sethi et al.
		Anti-gliadin +	Crypt hyperplasia		
W, 74 years old	Nivo	tTG-IgA +	Villous atrophy	GFD	Alsaadi et al.
	Ipi		Mucosal erosions	Corticosteroid (budesonide)	
			Inflammation in lamina propria		
			Intraepithelial lymphocytosis		
M, 79 years old	Pembro	tTG-IgA +	Villous atrophy	GFD (lack of adherence)	Arnouck et al.
		Anti-gliadin +	Lamina propria expansion, intraepithelial lymphocytes	Steroid (hydrocortisone)	
8 patients:	5 anti PD(L)1	tTG IgA: 8+/8	Only 6 of 8:	GFD: 7/8:	Badran et al.
2 W, 6 M	1 anti-CTLA4		inflammation, mucosal atrophy, mucosal ulcers or erosions (2/6), villous blunting (5/6), increased IELs (4/6), LP cellularity (6/6), surface erosion/ulceration (5/6)	- GFD alone: 5/8	
From 44 to 73 years old	2 combined			- GFD + steroid: 2/8	
				Steroid + infliximab: 1/8	

GFD, gluten-free diet, ICI, immune checkpoint inhibitor; IELs, intraepithelial lymphocytes; Ipi, Ipilimumab; LP, lamina propria, M, man; Nivo, Nivolumab, Pembo, pembrolizumab, PPI, proton pump inhibitors; tTG-IgA, tissue transglutaminase IgA antibodies; W, woman.

The first case reporting an ICI-CeD was published by Gentile et al. in 2013 with ipilimumab (9). A 62-year-old man with chemotherapy-naive, castration-resistant metastatic prostate cancer presented with refractory diarrhea despite prolonged high-dose corticosteroid treatment after receiving 3 doses of ipilimumab as part of a phase 3 clinical trial. The investigative work-up (anti-transglutaminase IgA+ and digestive endoscopy with crypt apoptosis and crypt distortion) confirmed CeD. The patient was initially treated with corticosteroid and infliximab, which was stopped after a gluten-free diet response.

Sethi et al. described the case of a 63-year-old Caucasian female diagnosed with metastatic adenocarcinoma of unknown primary origin, treated with a combination of carboplatin, paclitaxel, and pembrolizumab (10). The patient presented with diarrhea without blood, mild diffuse abdomen pain, and weight loss after starting pembrolizumab. The authors’ first hypothesis was colitis, but colonoscopy was negative. EGD with biopsy was then performed, which led to the diagnosis of CeD. Serological tests were inconsistent (anti-transglutaminase IgA − and anti-endomysium −, but anti-gliadin +). The patient was managed with strict gluten-free diet, which resolved her diarrhea.

Alsaadi et al. reported the case of a woman with metastatic renal carcinoma treated with six cycles of nivolumab (11) and then addition of ipilimumab. She presented vomiting and nausea and also diarrhea after the first injection. Upper endoscopy showed normal macroscopic mucosa but gastritis, duodenitis, and villous atrophy. Immunohistochemistry with anti-CD3 showed mildly increased intraepithelial T cells (20%–30% lymphocytes per hundred epithelial nuclei in the villi). Serum tissue transglutaminase IgA antibody level was elevated (12 U/ml). Treatment initially started with gluten-free diet, omeprazole, anti-emetics, and then with budesonide (corticosteroid) 9 mg per os daily. She stopped budesonide after 6 months.

Arnouck et al. (12) presented a case of a new-onset CeD after exposure to pembrolizumab. This 79-year-old melanoma patient developed loss of appetite and episodic diarrhea and a malabsorption. Stool studies and colonoscopy were normal. Capsule endoscopy revealed villous atrophy at the level of the second part of the duodenum. Duodenal biopsy shows lamina propria expansion, villous atrophy, and increased intraepithelial lymphocytes. Biologically, anti-gliadin and anti-transglutaminase Ig-A were in favor of CeD. Because of a lack of adherence of gluten-free diet, hydrocortisone (10 mg in the morning, 5 mg at night) was concurrently started.

The largest series was published by Badran et al., describing nine cases of ICI-duodenitis and eight cases of ICI-CeD (13), treated with anti-CTLA-4 or anti-PD-L1. All patients with duodenitis required immunosuppressive treatment. In ICI-CeD patients, only three of eight had immunosuppression: two received steroids before diagnosis of CeD and then were transitioned to gluten-free diet alone, resulting in disease remission. The other ones required steroid and infliximab. We have no information about the use of PPIs. The diagnosis of CeD was established by measurement of tTG IgA, and only six patients underwent endoscopy. Among these 8 patients, 5 benefited from ICI, with 3 complete responses and 2 partial responses. However, the authors do not detail if patients stopped or continued ICI after the ICI-CeD toxicity.

After reviewing the literature, the question remains open if ICI-CeD represents a *de novo* disease or a pre-existing asymptomatic CeD exacerbated by ICI. Indeed, no patient had a pre-ICI endoscopy or pre-ICI TG-IgA serology. Additionally, in the reported cases of ICI-CeD, no patient had a personal history of autoimmune disease. In our case, the patient had a history of type 1 diabetes mellitus. The prevalence of CeD is higher in these patients, with a mean prevalence of 8% (14) compared to 1% in the general population (11). Therefore, our patient was probably more likely to present with CeD. In addition, in the case series of Badran et al., 25% of patients with ICI-CeD had a family history of CeD. As tTG-IgA blood test presents an excellent sensitivity (around 96%) and specificity (approximately 91%) (8), we suggest that the tTG-IgA blood test might be used before ICI initiation in patients with an autoimmune history.

The physiopathology of ICI-induced CeD is largely unknown. Badran et al. described that ICI-CeD patients had increased intraepithelial CD3^+^ and CD8^+^ T cells and γδ T cells, compared to conventional CeD (13). These authors suggest that immune cell activation in the setting of ICIs results in unmasking of gluten sensitivity in genetically susceptible people, leading to expansion of previously self-reactive CD4^+^ T cells and subsequent CD8^+^ T cell-induced tissue destruction. Even with limited data, this hypothesis is biologically plausible and supported by similar mechanisms of other digestive toxicities (e.g., the large numbers of CD3^+^ and CD8^+^ infiltrate in ICI-induced hepatitis) (15).

Regarding treatment of CeD, the cornerstone is gluten-free diet, and is the only treatment currently recommended in classic CeD (8). Gluten-free diet was introduced in almost all cases reported of ICI-CeD, except for one patient in the case series of Badran et al. Treatments of digestive toxicities depend on severity of diarrhea according to common terminology criteria for adverse events (CTCAE v5.0). Grade 1 is defined as 4 stools over the baseline in 24 h, and symptomatic treatment (loperamide, rehydration, and electrolyte substitution) is advised. Grade 2 is defined as 4–6 stools over the baseline in 24 h and stool studies, diagnostic endoscopies, and treatment with steroids (1 mg/kg prednisone or equivalent) are indicated and ICI should temporarily be stopped. Severe diarrhea (grade 3: ≥7 stools/24 h over the baseline and grade 4 if associated with life-threatening consequences) requires hospitalization and high-dose IV corticosteroids such as methylprednisolone or dexamethasone. If no improvement is seen after 3–5 days, infliximab, a tumor necrosis factor-α (TNF-α) inhibitor, can be used. ICI is temporally stopped for grade 3 and permanently stopped for grade 4 (16, 17). On the basis of our literature review, about half of ICI-CeD patients only required gluten-free diet as a unique treatment. Patients receiving immunosuppressive treatments (mainly corticosteroids) were either those with a diagnosis established after treatment introduction, patients without sufficient efficacy of gluten-free diet (11), or patients with lack of adherence of gluten-free diet (11).

In conclusion, ICI-induced CeD is a diagnosis challenge. In the context of ICI, diarrhea can result from many causes, either gastrointestinal or not (thyroiditis, adrenal insufficiency). As the report of this toxicity is recent, ICI-induced CeD is probably underdiagnosed. This case and our literature review highlight the necessity of complementary investigation (digestive endoscopy with biopsy and tTG-IgA blood test) in patients with atypical digestive symptoms in the context of ICI treatment. This diagnosis challenge is clinically meaningful for the patient, as in most cases, a treatment of gluten-free diet may be sufficient.

## Data Availability Statement

The original contributions presented in the study are included in the article/supplementary material. Further inquiries can be directed to the corresponding author.

## Ethics Statement

The studies involving human participants were reviewed and approved by the Institutional Review Board of Institut Paoli Calmettes. Written informed consent was obtained from the participant for the publication of this case report (including all data and images).

## Author Contributions

JL and PR designed the research. JL and PR wrote the paper. All authors collaborated on the paper’s conception, reviewed the paper, and approved the final version of the article to be published.

## Funding

Our work is supported by Paoli-Calmettes Institute, Aix-Marseille University, and Fondation ARC (SIGN’IT program).

## Conflict of Interest

The authors declare that the research was conducted in the absence of any commercial or financial relationships that could be construed as a potential conflict of interest.

## Publisher’s Note

All claims expressed in this article are solely those of the authors and do not necessarily represent those of their affiliated organizations, or those of the publisher, the editors and the reviewers. Any product that may be evaluated in this article, or claim that may be made by its manufacturer, is not guaranteed or endorsed by the publisher.

## Abbreviations

CeD, celiac disease; EGD, esophagogastroduodenoscopy; GERD, gastroesophageal reflux disease; GFD, gluten-free diet; GI, gastrointestinal; ICI, immune checkpoint inhibitor; PPI, proton pump inhibitors; tTG-IgA, tissue transglutaminase IgA antibodies.
